# Unseen dualities: underdiagnosis of substance use disorders in borderline personality disorder

**DOI:** 10.3389/fpsyt.2025.1539611

**Published:** 2025-03-25

**Authors:** Jana Nabel, Sebastian Bertele, Britta Stapel, Nicole Scharn, Kai G. Kahl

**Affiliations:** Department of Psychiatry, Socialpsychiatry and Psychotherapy, Hannover Medical School, Hannover, Germany

**Keywords:** borderline personality disorder, substance use disorder, alcohol use disorder, cannabis use disorder, prevalence analysis, medical record, SCID

## Abstract

**Introduction:**

Borderline personality disorder (BPD) patients exhibit high rates of co-occurring mental disorders. Though literature reports varying prevalence of substance use disorders (SUD) in BPD, they are frequent with prevalence rates of approximately 45%. This study examines the 12-month prevalence of SUDs in a German sample of BPD patients by semi-structured interviews and compared to medical records.

**Methods:**

N=126 BPD patients were included. Twelve-month SUD prevalence was obtained by semi-structured clinical interview (SCID) and compared to reported prevalence in the general population and to diagnoses from medical records.

**Results:**

Mean age of the sample was 37.5 (SD ± 11.5) years and N=61 (48%) patients were female. Compared to the general population, increased 12-month prevalence based on SCID was found for alcohol abuse (22.2%, +7.9-times), alcohol dependence (17.5%, +5.6-times), cannabis abuse (15.1%, +30.2-times), cannabis dependence (19.0%, +31.7-times), sedative abuse (3.2%, +4.6-times) and sedative dependence (3.2% +4.6-times). N=43 (34.1%) patients presented at least one diagnosis of substance abuse and N=43 (34.1%) presented at least one diagnosis of substance dependence. N=51 (40.1%) patients were diagnosed with at least one substance use or dependence disorder. On average, prevalence based on SCID exceeded prevalence obtained from medical records. Particularly, alcohol abuse (3.5-times), cannabis abuse (2.4-times) and dependence (2.2-times), and sedative- and stimulant abuse (both no diagnosis in medical record *vs*. 3.2% in SCID) were underrecognized. Furthermore, concordance rates between diagnoses based on medical record and SCID were below 30% for all substances.

**Conclusion:**

Our data confirm high prevalence of SUDs in BPD patients. Of note, medical records underrecognized alcohol abuse and cannabis abuse and dependence. Substance abuse and dependence are primary risk factors of suicidal behaviors and completed suicide. SUDs have been shown to be amenable to psychotherapeutic interventions. Therefore, careful diagnosis of SUD by e.g. expert structured interviews and integration of SUDs in a multimodal treatment plan is recommended.

## 1 Introduction

Consumption of psychoactive substances is common in the general population and their use and abuse is associated with a significant burden of disease worldwide (1). Current literature reports the highest number of disability-adjusted life years (DALY) and death for tobacco use with alcohol consumption and illegal drug use following in second and third place, respectively (2). Compared to the general population, use of psychoactive substances is more frequent in clinical samples. Particularly in the context of psychiatric disorders, it constitutes a common problem and substance use disorders (SUD) are frequent (3). In particular, alcohol use disorder is the most prevalent SUD in every category of psychiatric disorder, including personality disorders (3). Dual disorder patients, i.e. patients that are characterized by the co-occurrence of two syndromes, have a higher risk of all-cause mortality (4) and a less favorable prognosis due to a reduced effectiveness of pharmacological as well as psychosocial and psychotherapeutic interventions (5, 6). Patients with severe mental illness and SUD have higher rates of hospitalization and an increased number of contacts with psychiatric emergency departments compared to patients without SUD (7).

Among the personality disorders that commonly co-occur with SUD, borderline personality disorder (BPD) has received particular attention because of its complex clinical presentation. The symptoms of borderline personality disorder (BPD) can be grouped in three principles domains: emotional dysregulation, inconsistent identity and disturbed interpersonal functioning (8). Epidemiological studies suggest prevalence rates between 0.7-2.7% in non-clinical samples (9, 10) In clinical samples, BPD constitutes the most frequent personality disorder that affects up to 10% of psychiatric outpatients and up to 25% of inpatients (11). BPD is frequently associated with the co-occurrence of additional psychiatric disorders, with mood and anxiety disorders, non-borderline personality disorders, and SUD being the most prevalent (12). Vice versa, personality disorders are frequently diagnosed in patients that are treated for an SUD and a prevalence for BPD between 5-22% has been reported in this patient population (13, 14). This is in line with findings from a dedicated review article reporting a prevalence of 22.1% for current BPD among cases with a current SUD across studies (15). Similarly, high prevalence for any SUD has been described in patients with BPD, with dedicated cross sectional studies reporting rates between 19-87% for any SUD and 24-66% for an alcohol-related SUD (16). While diagnostic criteria for BPD and SUDs intersect, the overlap does not account for the pronounced observed co-occurrence of BPD and SUDs and BPD rates in SUD patients remain high even when substance related characteristics are not included in the diagnosis (17, 18).

Compelling evidence suggests an adverse impact of the co-occurrence of BPD in patients with SUDs (19). By comparison these patients are younger, more frequently female, and less frequently employed compared to SUD patients without BPD. Additionally, they display high rates of mood disorders and low levels of functioning (20, 21). Conversely, studies regarding the impact of a co-occurring SUD on BPD symptomatology and outcome are less frequent and yielded inconsistent results. Prior studies suggest that patients with co-occurring BPD and past or current SUD report higher impulsivity than those without SUD (22), although some found no significant differences in BPD symptoms (23, 24). However, suicide and self-harm thoughts are more common in BPD patients with SUD (25, 26), and their remission rates are lower. SUD is also a significantly worse prognostic factor than other co-occurring disorders, such as PTSD, anxiety, or mood disorders (27).

Previous studies have addressed prevalence rates of SUDs in BPD patients. However, most of these studies only distinguished between alcohol abuse and dependence (DSM-IV, ICD-10), while other substances have been commonly grouped as drug abuse and dependence (again DSM-IV, ICD-10).

In the present manuscript we assessed 12-month prevalence rates of substance abuse and dependence (by ICD-10 as we assessed a German population), and substance use disorder as defined by DSM-5 of individual substances in a sample of BPD patients by semi-structured clinical interview (SCID DSM-5). We compared obtained prevalence to data from the general population and to diagnoses obtained from medical records. To our knowledge a comparison of SCID diagnoses and medical records, evaluating potential over- or underdiagnosis of SUD in BPD has not been performed before.

## 2 Methods

### 2.1 Study design and participants

The present study followed the principals of the Declaration of Helsinki and was approved by the local ethics committee at Hannover Medical School. All participants gave their consent before study inclusion. Structured clinical interviews and follow-up data were acquired between September 2021 and April 2023 (in one telephone interview session).

Patients with a prior diagnosis of borderline personality disorder that were previously treated at the ward for dialectical behavior therapy of the Department of Psychiatry, Social psychiatry, and Psychotherapy at Hannover Medical School (but not in a structured follow up) were contacted by telephone. Inclusion criteria were as follows: a diagnosis of BPD, an age of 18 years or older, treatment for BPD in MHH within the past year, and sufficient German langue skills to understand the consent form and to participate in the SCID. Severe cognitive disorders (known IQ <70; previous clinical diagnosis; severe intoxication) were considered an exclusion criterion. Off 324 patients contacted, N=126 patients with BPD agreed to partake in and were included in the study.

To assess the 12-month prevalence of mental and behavioral disorders due to psychoactive substance use (ICD-10-CM codes F10-F19) the German version of the semi-structured clinical interview (SCID) for Diagnostic and Statistical Manual-5 (DSM-5) was performed (28, 29). In particular, module E (Substance Use Disorders) with items E1-10 (Alcohol Use Disorders in the past 12 month) and E11-E36 (Nonalcohol Substance Use Disorders in the past 12 month) of the SCID-5 was applied by telephone interview by a trained research assistant under supervision (J.N.; supervision N.S. and K.G.K). Additionally, data regarding all prior psychiatric diagnoses, including substance abuse and dependence disorders were obtained from patient’s medical records by J.N.

We obtained ICD-10 SCID-validated diagnoses by matching ICD-10 criteria to the SCID-5-CV items. For harmful use that included the consumption with loss of control, as well as at least one item for social, psychological or physical harm (items E6-10 for alcohol and E28-32 for nonalcohol substances). For dependence we required 3 or more symptoms correlating with craving (items E3 for alcohol; E25 for nonalcohol substances), loss of control over consumption (E2-3 and E24-25), continued use despite harm (E6-7, E9-10; E28-29, E30-31), disregard of other activities in favor of substance use (E4, E8; E26, E30), development of tolerance (E11; E33), and withdrawal symptoms (E12, E34).

#### 2.1.1 Sample characteristics and psychiatric comorbidities

The mean age of BPD patients in the present sample was 37.5 (SD ± 11.5) years and N=61 (48%) were female. Based on medical record, prevalence of psychiatric comorbidity was high, with N=117 (93%) patients having at least one other F-diagnosis than BPD and F10 diagnoses. Prevalence of F-diagnoses based on medical record of the total sample as well as grouped by sex are summarized in **Table 1**.

**Table 1:** Psychiatric comorbidities in male and female patients with BPD based on medical record.

ICD code	Total	Female	Male	Comparison based on sex
F-20-F29 (N, %) Schizophrenia, schizotypal, delusional, and other non-mood psychotic disorders	4, 3.2%	2, 3.3%	2, 3.1%	χ²(1)=.004, φ=-.006, *p*=.949
F-30-F39 (N, %) Mood (affective) disorders	107, 84.9%	52, 85.2%	55, 84.6%	χ²(1)=.010, φ=-.009, *p*=.921
F40-F48 (N, %) Anxiety, dissociative, stress-related somatoform and other nonpsychotic mental disorders	76, 60.3%	33, 54.1%	43, 66.2%	χ²(1)=1.911, φ=.123, *p*=.167
F50-F59 (N, %) Behavioral syndromes associated with physiological disturbances and physical factors	25, 19.8%	15, 24.6%	10, 15.4%	χ²(1)=1.677, φ=-.115, *p*=.195
*F60-F69 (N, %) Disorders of adult personality and behavior	22, 17.5%	9, 14.8%	13, 20.0%	χ²(1)=.601, φ=.069, *p*=.438
F90-F98 (N, %) Behavioral and emotional disorders with onset usually occurring in childhood and adolescence	15, 11.9%	7, 11.5%	8, 12.3%	χ²(1)=.021, φ=.013, *p*=.885

N-numbers and point prevalence rates of psychiatric comorbidities based on medical record in the total sample as well as in female and male patients with borderline personality disorder. Group differences between male and female patients were assessed by use of Chi-Square test. *excluding BPD. ICD, international statistical classification of disease and related health problems.

### 2.2 Statistical analysis

Statistical analyses were performed using SPSS 28 (IBM, Armonk, NY, USA) and R 4.4.3 (Trophy Case; R Core TeamR Foundation for Statistical Computing, Vienna, AUSTRIA). Point prevalence rates based on SCID and medical record were calculated and 95% confidence intervals (CI) were computed using the Clopper-Pearson method. Shapiro-Wilk test was used to test for normal distribution of continuous data. Group comparisons of categorical data were carried out by use of Chi-Square test and Mann-Whitney U test was performed for comparison of continuous data. Two-tailed p-values are depicted and p≤.050 was considered statistically significant. Concordance statistics were calculated using Kendall’s tau.

## 3 Results

### 3.1 Comparison of 12-month prevalence rates of mental and behavioral disorders due to psychoactive substance use in BPD patients and the general population

Comparison of point prevalence rates of the three most frequently used substances in BPD patients to literature data from the German general population are depicted in **Table 2** (30). The point prevalence of SUDs in the study’s BPD sample was significantly higher than reported data in the general population. This applied to both male and female patients.

**Table 2:** Comparison of substance abuse and dependence in BPD patients with data from the general population.

	Total		Female		Male	
ICD code	BPD	General population *	BPD	General population *	BPD	General population *
F10.1 Alcohol abuse	22.2% [15.3; 30.5]	2.8% [2.4; 3.3]	21.3% [11.9; 33.7]	1.5% [1.1; 2.0]	23.1% [13.5; 35.2]	4.0% [3.3; 4.9]
F10.2 Alcohol dependence	17.5% [11.3; 25.2]	3.1% [2.7; 3.6]	19.7% [10.6; 31.8]	1.7% [1.4; 2.1]	15.4% [7.6; 26.5]	4.5% [3.7; 5.3]
F12.1 Cannabis abuse	15.1% [9.3; 22.5]	0.5% [0.4; 0.7]	19.7% [10.6; 31.8]	0.4% [0.2; 0.6]	10.8% [4.4; 20.9]	0.7% [0.5; 1.1]
F12.2 Cannabis dependence	19.0% [12.6; 27.0]	0.6% [0.4; 0.9]	21.3% [11.9; 33.7]	0.3% [0.2; 0.5]	16.9% [8.8; 28.3]	1.0% [0.6; 1.5]
F13.1 Sedative abuse	3.2% [0.9; 7.9]	0.7% [0.5; 0.9]	3.3% [0,4; 11.3]	0.5% [0.3; 0.8]	3.1% [0.4; 10.7]	0.8% [0.5; 1.2]
F13.2 Sedative dependence	3.2% [0.9; 7.9]	0.7% [0.5; 1.1]	3.3% [0.4;11.3]	0.6% [0.4; 0.9]	3.1% [0.4; 10.7]	0.9% [0.5; 1.5]

12-month prevalence rates and 95% confidence intervals are depicted. *Data from the general population were extracted from (30). ICD, international statistical classification of disease and related health problems.

Alcohol related disorders are the second most common SUD (or, for that matter, dependence and harmful use by ICD-10 criteria) in the general population. In this study’s BPD sample, we found a 7.9-times (female: 14.2-times, male: 5.8-times) higher prevalence of alcohol abuse while prevalence of alcohol dependence showed a 5.6-times increase (female: 11.6-times, male: 3.4-times) when compared to the general German population. Overall, N=50 (39.7%) BPD patients presented with an alcohol use disorder based on SCID.

Prevalence for cannabis related disorders was less than one percent in the general population (30). The present data indicate significantly higher point prevalence in BPD patients, with cannabis abuse exceeding data from the general population by 30.2-times (female: 49.3-times, male: 15.4-times) and cannabis dependence by 31.7-times (female: 71.0-times, male:16.9-times). Indeed, cannabis use disorders were the nearly as prevalent as alcohol use disorders in the present BPD sample, with N=43 (34.1%) displaying cannabis use or dependence disorders.

Concurrent with cannabis use disorders, prevalence of sedative abuse and dependence in the general population were less than one percent (30). In contrast, in this study’s sample point prevalence for sedative abuse fell above 3% for the total as well as the male and female subgroups. Sedative abuse was 4.6-times higher in the complete sample (female: 6.6-times, male: 3.9-times) than in the general population. Further, sedative dependence disorder was more frequent (4.6-times) in BPD patients compared to the general population (female: 5.2-times, male: 3.4-times). The overall 12-month prevalence rate for a sedative use disorder was 6.3% (N=8) in this study’s sample.

Based on data obtained from SCID, N=58 (46.0%) of BPD patients in the present sample had at least mild SUD (at least 2 DSM-5 criteria), while at least one diagnosis of moderate to severe SUD (four or more DSM-5 criteria) was found in N=41 (32.5%) of patients.

### 3.2 Comparison of prevalence rates of substance use disorders in male and female BPD patients

Male and female BPD patients in the present sample did not differ significantly with regard to mean age (female: 36.2 ± 11.1 years, male: 38.5 ± 11.9 years, *U*=1750.5, *Z*=-1.133, p=.257) or psychiatric comorbidities (**Table 1**).

No significant differences regarding prevalence rates of specific SUD determined by SCID were found in female compared to male BPD patients (**Table 3**). Additionally, female and male BPD patients did not differ significantly regarding frequencies of at least one diagnosis of any dependence or harmful use (**Table 3**).

**Table 3:** Similar prevalence rates of substance abuse and dependence disorders in female and male BPD patients based on SCID.

ICD-10 code	Female (N=61)	Male (N=65)	Statistic
F10.1, Alcohol abuse (N, %)	13, 21.3%	15, 23.1%	χ²(1)=.001, φ=.002, *p*=.981
F10.2, Alcohol dependence (N, %)	12, 19.7%	10, 15.4%	χ²(1)=.159, φ=.036, *p*=.690
F11.1, Opioid abuse (N, %)	0, 0.0%	0, 0.0%	χ²(1)=.127, φ=.032, *p*=.722
F11.2, Opioid dependence (N, %)	0, 0.0%	0, 0.0%	χ²(1)=.127, φ=.032, *p*=.722
F12.1, Cannabis abuse (N, %)	12, 19.7%	7, 10.8%	χ²(1)=1.315, φ=.102, *p*=.252
F12.2, Cannabis dependence (N, %)	13, 21.3%	11, 16.9%	χ²(1)=.160, φ=.036, *p*=.689
F13.1, Sedative abuse (N, %)	2, 3.3%	2, 3.1%	χ²(1)=.000, φ=.000, *p*=1.000
F13.2, Sedative dependence (N, %)	2, 3.3%	2, 3.1%	χ²(1)=.000, φ=.000, *p*=1.000
F15.1, Stimulant abuse (N, %)	2, 3.3%	2, 3.1%	χ²(1)=.000, φ=.000, *p*=1.000
F15.2, Stimulant dependence (N, %)	3, 4.9%	2, 3.1%	χ²(1)=.005, φ=.006, *p*=.942
F16.1, Hallucinogen abuse (N, %)	0, 0.0%	0, 0.0%	χ²(1)=.127, φ=.032, *p*=.722
F16.2, Hallucinogen dependence (N, %)	0, 0.0%	0, 0.0%	χ²(1)=.127, φ=.032, *p*=.722
F18.1, Inhalant abuse (N, %)	0, 0.0%	1, 1.5%	χ²(1)=.000, φ=.000, *p*=1.000
F18.2, Inhalant dependence (N, %)	1, 1.6%	2, 3.1%	χ²(1)=.000, φ=.000, *p*=1.000
At least one substance abuse disorder (N, %)	21, 34.4%	22, 33.8%	χ²(1)=0, φ=0, p=1
At least one substance dependence disorder (N, %)	24, 39.3%	19, 29.2%	χ²(1)=1.017, φ=0.09, p=0.313
At least one substance abuse or dependence disorder (N, %)	26, 42.6%	25, 38.5%	χ²(1)=0.086, φ=0.026, p=0.769

N-numbers and 12-month prevalence rates based on structured clinical interview for DSM disorders (SCID) are depicted. Group difference between male and female patients with borderline personality disorder were computed using Chi-Square test. ICD, international statistical classification of disease and related health problems.

### 3.3 Comparison of prevalence rates of mental and behavioral disorders due to psychoactive substance use based on medical record and SCID

**Figure 1:**
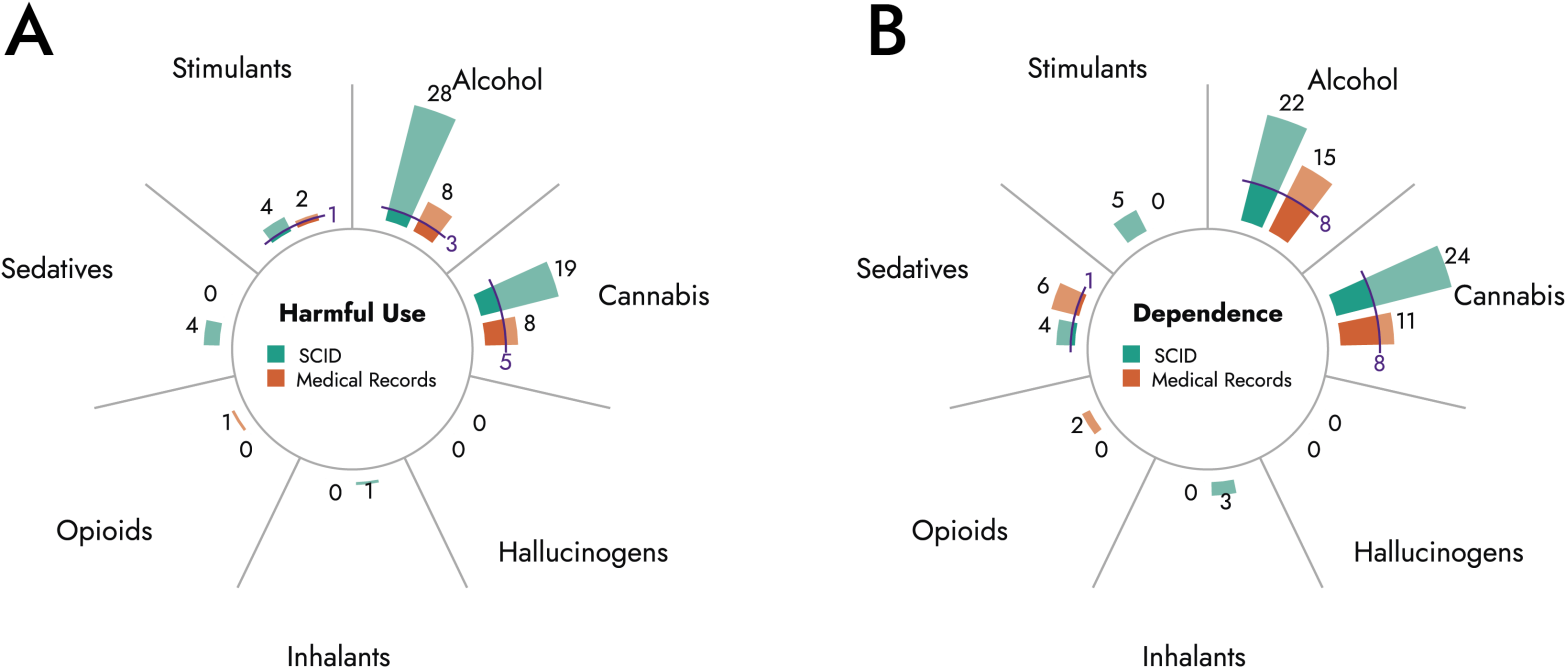
Comparison of substance abuse and dependence diagnoses based on SCID and medical record. Diagrams depict total number of cases diagnosed with harmful use **(A)** or dependence **(B)** of indicated substances based on SCID (green) or medical records (orange). Blue lines indicate cases with matching diagnoses in medical record and SCID.

**Figures 1A, B** as well as **Table 4** compare numbers of substance abuse and substance dependence cases based on SCID diagnoses to case numbers obtained from medical records. Alcohol abuse and dependence and cannabis abuse and dependence were the most frequent ICD-10 diagnoses in medical records and SCID. However, present data suggest an underdiagnosis of SUD in patients with BPD. On average, prevalence rates based on SCID exceeded prevalence rates obtained from medical record. Particularly, alcohol abuse (3.5-times) and dependence (1.5-times), cannabis abuse (2.4-times) and dependence (2.2-times), sedative abuse (no diagnosis in medical record *vs*. 3.2% in SCID), and stimulant abuse (2.0-times) were underrecognized in medical records. Conversely, frequencies of opioid abuse and dependence and sedative dependence were low based on medical record as well as based on SCID.

**Table 4:** Discrepancies between substance abuse and substance dependence diagnoses based on SCID and medical record.

ICD-10 code	Total Cases	SCID Cases	Medical Record Cases	Concordance Rate	Kendall’s Tau	p-value
F10.1, Alcohol abuse (N)	33	28	8	9.1%	.347	.2847
F10.2, Alcohol dependence (N)	29	22	15	27.6%	.096	<.050
F11.1, Opioid abuse (N)	1	0	1	0.0%	–	
F11.2, Opioid dependence (N)	2	0	2	0.0%	–	
F12.1, Cannabis abuse (N)	22	19	8	22.7%	.345	<.050
F12.2, Cannabis dependence (N)	27	24	11	29.6%	.423	<.050
F13.1, Sedative abuse (N)	4	4	0	0.0%	–	
F13.2, Sedative dependence (N)	9	4	6	11.1%	.172	.0540
F15.1, Stimulant abuse (N)	5	4	2	20.0%	.339	<.050
F15.2, Stimulant dependence (N)	5	5	0	0.0%	–	
F16.1, Hallucinogen abuse (N)	0	0	0	–	–	
F16.2, Hallucinogen dependence (N)	0	0	0	–	–	
F18.1, Inhalant abuse (N)	1	1	0	0.0%	–	
F18.2, Inhalant dependence (N)	3	3	0	0.0%	–	

N-numbers for total cases (i.e. diagnosis based on SCID and/or medical record), SCID cases (i.e. diagnosis based on SCID) and medical record cases (i.e. diagnosis based on medical record) are shown. Concordance rate in percent indicates the percentage of cases with a corresponding diagnosis based on SCID and medical record. Kendall’s Tau is listed where calculatable; CIs were incalculable due to low frequencies. ICD, international statistical classification of disease and related health problems; SCID, structured clinical interview for DSM disorders.

Furthermore, concordance rates between diagnoses based on medical records and SCID were low (**Table 4**, **Figures 1A, B**). The highest concordance rates were observed for cannabis dependence (29.6%) and alcohol dependence (27.6%), while diagnoses for harmful use of sedatives or stimulants showed no overlap between medical records and SCID.

## 4 Discussion

In the present study we describe increased 12-month prevalence rates of SUDs in patients with BPD compared to the general population. Our data are in line with previous studies describing an elevated prevalence for SUDs in BPD patients compared to the general population. In contrast to studies by others, we did not observe sex-specific effects on SUD prevalence in the present sample. Additionally, we report an underrepresentation of SUD diagnoses in medical records of BPD patients compared to data based on SCID-5. While DSM-5 criteria for substance use disorders that underly SCID differ from ICD-10 criteria that underly German medical records, and are more prone to diagnose a patient with SUD as even fulfilling two of eleven symptoms classifies as mild SUD, the differences in the current sample are too large to be explained by this effect.

### 4.1 Increased prevalence of SUDs in patients with BPD compared to the general population

The present study highlights a significantly higher 12-month prevalence for SUDs, particularly mild SUDs, in BPD patients compared to the general public. In this regard, our findings are in line with previous works that consistently report high co-occurrence rates for 12-month and lifetime prevalence for SUDs in BPD. A meta-analysis by Trull and colleagues that only included studies utilizing structured interviews for SUD diagnosis, found an average co-occurrence rate of approximately 45% for at least one current SUD in BPD patients across settings (15), which is slightly lower than the observed prevalence of 46% for any SUD in the present sample. In the general population alcohol is the most frequently used substance, excluding nicotine, by some margin (30). Prior studies in the context of BPD yielded heterogenous results concerning prevalence of alcohol use disorders. Trull and colleagues report high prevalence rates of approximately 46% for current alcohol use disorder in BPD patients across settings (15), while a second systemic review article reported a 12-month prevalence for alcohol use disorders between 13% and 31%, based on 16 studies that used structured (N=15) or unstructured (N=1) interviews for SUD diagnosis (31). Our data indicate a 12-month prevalence of 25% for alcohol use disorder based on SCID.

Contrarily to data from the general population and to prior studies in BPD patients, cannabis use disorders were more prevalent than alcohol-related disorders in the present sample (15, 30). There are fewer studies that assess cannabis use disorders in BPD patients than those targeted at comorbidity of BPD and alcohol-related disorders. This can be attributed to the fact that the majority of respective do not distinguish between different substances, but rather assessed drug use disorders in aggregate. The underlying rational for the combined analysis of nonalcoholic substances as one category might relate to the relatively low prevalence and subsequent limited sample sizes. However, one publication based on data from the National Institute on Alcohol Abuse and Alcoholism’s (NIAAA) NESARC study and that analyzed lifetime prevalence of substance use and dependence in BPD patients in relation to specific substances, reported a lifetime prevalence for cannabis use disorder of 31% in a sample of 1,030 BPD patients (32). This aligns with the 12-month prevalence of cannabis use disorder in this sample according to medical records whereas cannabis use disorders identified through SCID show a slight under representation in our sample (25.2%). While we can only speculate about the underlying reasons, several factors might be considered. Cannabis use has been increasing in the general population in recent years, owing to the progressive legalization and/or decriminalization in several countries, including Germany (33–35). Additionally, research suggests that coping motives as well as conformity motives are strongly associated with borderline features for both alcohol and cannabis (36). Therefore, particularly conformity motives may contribute to increased cannabis use in BPD patients given the increasing popularity of the substance in the general population. Further, studies have shown that cannabis use may exert anxiolytic effects, reduce impulsivity and self-harming behavior, and mitigate stress reactivity—features frequently observed in BPD (37–39).

Sedative use disorder was reported to have a lifetime prevalence of 8.4% in BPD patients (32). To the best of our knowledge, data regarding 12-month prevalence rates for sedative use disorders in this patient population are lacking to date. In the current sample, 12-month prevalence rates were with 4.8% markedly higher than in the general population.

Overall, our data are in line with previous studies reporting significantly increased rates of substance abuse and dependence in BPD patients compared to the general public.

### 4.2 Effect of sex

In the general population, SUDs occur with a higher frequency in men compared to women (30). Similarly, studies assessing potential differences regarding frequency of SUDs in male and female patients with BPD have indicated higher prevalence rates for alcohol use disorders and substance use disorders in men compared to women (40–42). Contrarily, in the present sample no sex-specific differences in 12-month prevalence rates were observed for any substance, and additionally male and female patients did not differ significantly regarding the frequency of at least one substance use or dependence disorder. Given the overall higher rates of SUDs, particularly of alcohol-related disorders, in men in the general population, most SUDs appear to be stronger overrepresented in female BPD patients compared to males. Considering the overall higher risk of BPD-individuals for SUDs reported in literature (43).

### 4.3 Importance of recognizing SUDs in BPD patients

While previous research regarding the impact of a co-occurring SUD on BPD symptomology and trajectory yielded inconsistent results, there is some evidence that indicates higher levels of suicidality and self-harm and less frequent remission from BPD in the context of any SUD (44, 45). Additionally, a co-occurring SUD was found to be associated with an increased likelihood of high-risk sexual behavior reflected by higher rates of sexual transmitted diseases in BPD patients (46).

Further, patients with co-occurring BPD and SUD might show decreased adherence to therapy when only one disorder is taken into account, which is reflected by increased dropout rates in dual patients (47). Likely due to the incompability of ongoing substance use and effective psychotherapy or physical inability to attend psychotherapy due to intoxication. Also, craving for substances can significantly impact treatment adherence in more intensive settings like day-hospitals or inpatient-treatments. Due to lack of evidence, this does remain speculative, however.

Given the described adverse effects of SUDs surprising little literature exists that covers treatment approaches for co-occurring BPD and SUD (48). Although psychotherapy is considered the first-line treatment option for BPD and also plays part in SUD treatment, studies assessing the efficacy of psychotherapy in dual disorder patients are scarce and limited by small sample sizes and variable outcomes. However, there is some evidence that structured integrative care, particularly dialectic behavior therapy for SUD (DBT-SUD) and to a lesser extent for dynamic deconstructive psychotherapy (DDP) are associated with beneficial outcomes (48). Pharmacologically there is little evidence for BPD but a co-occurring SUD might need substitution or can benefit from medication (e.g. bupropione for nicotine use or acamprosate for alcohol use disorders or naltrexone to reduce craving). Therefore, it is of importance to adequately diagnose SUDs in BPD patients to enable them to receive optimized treatment. However, data from this study suggests that in clinical practice the co-occurrence of both disorders is often overlooked – particularly in the case of cannabis use disorders.

An underdiagnosis of SUDs appears to be common, which is reflected in our data showing significantly lower prevalence rates for most SUDs based on data obtained from medical records compared to SCID.

In this regard, particularly substance abuse was more often not recognized in medical records compared to substance dependence. This may be due to the ICD-10 criteria requiring negative physical health consequences for the diagnosis of substance abuse whereas the adapted SCID-5 criteria, based on DSM-5, do not require this criterion for the definition of mild SUD. In clinical practice that may lead to underestimating the impact of a less severe SUD on the overall treatment as the substance abuse does not appear in diagnoses of discharge summaries and medical histories but may get lost in texts describing the patient’s behaviors. Considering the relevant negative impact of substance abuse on both physical and mental health, taking special care to test for even mild forms of substance abuse or applying DSM-5 criteria for the actual treatment, could improve overall outcomes. In addition, concordance rates between diagnoses based on medical records and SCID were low, suggesting that either at the time of medical record’s creation patients exhibited markedly different substance use behaviors or diagnostic criteria applied in clinical setting differed from the gold-standard criteria used in SCID. Another possible explanation was “carry-on” of old diagnoses in medical records that patients had had in previous treatments.

We are unaware of studies that focused on the accuracy of medical records regarding SUD diagnoses in BPD patients and similarly, we could not identify research that investigated potential reasons for the under-recognition of SUDs in this patient population. However, literature relating to dual patients with schizophrenia and SUDs suggest that under-reporting amongst patients is common (49). This is in line with a meta-analysis including studies that compared substance use based on self-report to laboratory tests in a general adult mental health setting (50). This study reported that laboratory test yielded positive results in 27% of patients that denied use of any substance with (50). Further, the authors also proposed that using structured interviews regarding substance use might yield more accurate results than other methods of clinical assessment. From a clinical perspective, factors such as shame about the use or abuse of psychoactive substances as well as a fear of being required to be abstinent from a substance they have found to be helpful in mitigating fear- and anxiety-related symptoms. They may, in that context, not even consider the use of the substance as “abuse” or “dependence”. Lastly, reporting regular use of substances and a diagnosis of a SUD limit access to psychotherapeutic treatments – abstinence may be a prerequisite for therapy or the insurance company may refuse reimbursement for patients consuming psychoactive substances. Patients, therefore, have many motives beyond the dysfunctional or stereotype to avoid mentioning problematic substance use.

### 4.4 Limitations

The present study has several limitations that should be taken into account when interpreting the results. First, we did not record reasons for non-participation. Therefore, we cannot exclude the possibility of some bias regarding the present study sample, i.e. patients with problematic substance use might be less likely to agree to participate. Second, we did not perform a SCID for other psychiatric disorders other than SUDs. Therefore, we were unable to assess if the observed underdiagnosis of SUDs in medical records extended to other mental disorders or whether the observed discrepancy was specific for SUDs. Third, while studies suggest that SCID is a more reliable method for diagnosis of SUDs in patients with severe mental illnesses than other interview- or self-report instrument, SCID does rely on the accuracy of the information given by the patient. Therefore, we cannot exclude some inaccuracies regarding the reported 12-month prevalence rates.

## 5 Conclusion

Our study confirms high rates of SUDs in patients with BPD compared to the general population. Of note, particularly cannabis use and dependence was high in the present sample, exceeding prevalence rates for alcohol-related disorders. In contrast to prior studies, prevalence rates for any SUD did not significantly differ between sexes. Based on comparison with data retrieved from medical records, SUDs were often not adequately diagnosed in this patient population. While we can only speculate about the underlying reasons for this observed discrepancy, it is important to note the potential resulting adverse consequences for the individual patient but also for the health care system (51, 52). Given the unfavorable outcomes of BPD patients with co-occurring SUDs (44, 45, 53), adequate diagnostic evaluation and consequent referral to indicated therapies is of paramount importance to not only ensure short-term adherence but a favorable long-term outcome. Therefore, clinical professionals treating patients with BPD should encourage patients to be open about their substance use and not hesitate to diagnose and educate about SUDs to reduce stereotypes, encourage multi-disorder treatment and help their patients avoid long-term adverse health events.

## Data Availability

The raw data supporting the conclusions of this article will be made available by the authors, without undue reservation.
